# (1*R*,3*S*,8*R*)-3,7,7,10-Tetra­methyl­tri­cyclo­[6.4.0.0^1,3^]dodec-9-en-11-one

**DOI:** 10.1107/S1600536813034041

**Published:** 2013-12-21

**Authors:** Abdoullah Bimoussa, Aziz Auhmani, My Youssef Ait Itto, Jean-Claude Daran, Auhmani Abdelwahed

**Affiliations:** a Laboratoire de Physico-Chimie Moléculaire et Synthése Organique, Département de Chimie, Faculté des Sciences, Semlalia BP 2390, Marrakech 40001, Morocco; b Laboratoire de Chimie de Coordination, 205 route de Narbonne, 31077 Toulouse Cedex 04, France

## Abstract

The absolute configuration of the title compound, C_16_H_24_O, has been deduced from the chemical pathway. The six-membered ring has a roughly half-chair conformation with the quaternary C atom as the flap. The seven-membered ring displays a chair conformation. In the crystal, there is a weak C—H⋯O hydrogen bond between the methyl­ene group of the cyclo­propane ring and the carbonyl group of a screw-axis-related mol­ecule, which builds up a zigzag-like chain along the *b-*axis direction.

## Related literature   

For related structures, see: Benharref *et al.* (2012 ▶); Gassman & Gorman (1990 ▶); Lassaba *et al.* (1997 ▶). For ring puckering analysis, see: Cremer & Pople (1975 ▶). For structural discussion, see: Evans & Boeyens (1989 ▶); Spek (2009 ▶). For chemical properties, see: Auhmani *et al.* (2002 ▶); Danyang *et al.* (2007 ▶); Tetsuhiro *et al.* (2003 ▶).
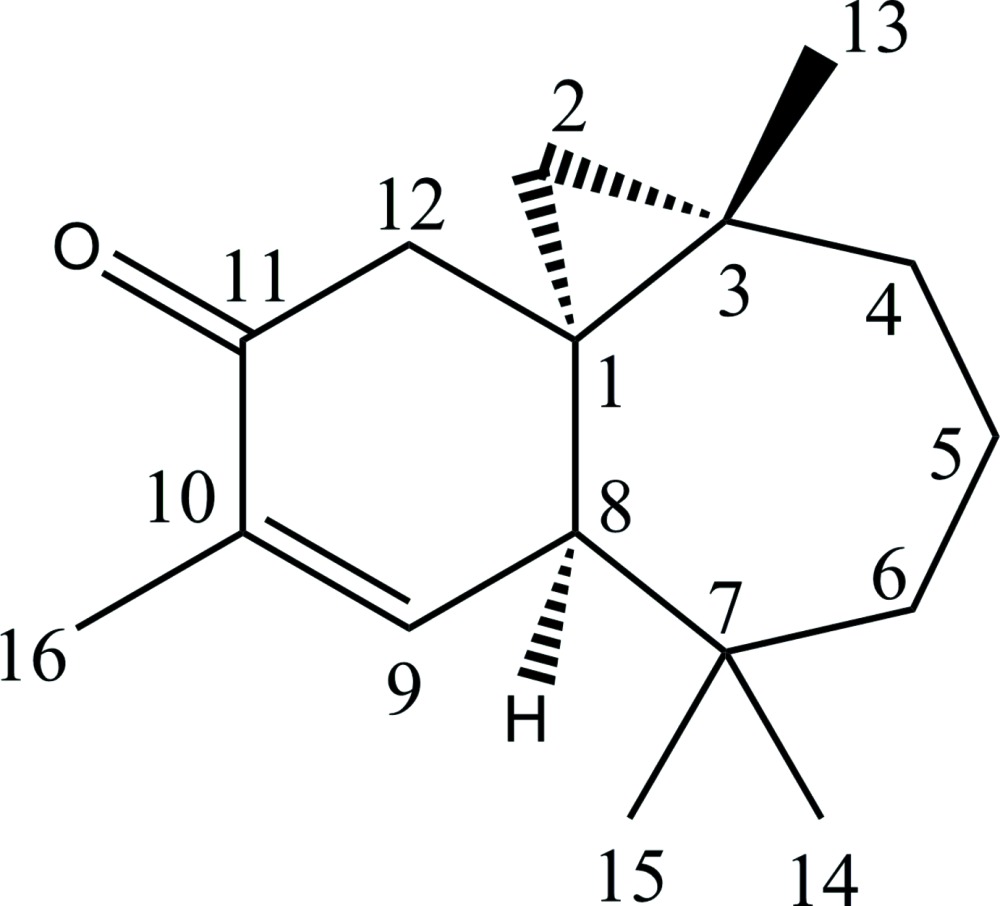


## Experimental   

### 

#### Crystal data  

C_16_H_24_O
*M* *_r_* = 232.35
Monoclinic, 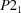

*a* = 6.4379 (2) Å
*b* = 7.8889 (3) Å
*c* = 13.5122 (5) Å
β = 97.430 (2)°
*V* = 680.49 (4) Å^3^
*Z* = 2
Mo *K*α radiation
μ = 0.07 mm^−1^
*T* = 180 K
0.38 × 0.23 × 0.08 mm

#### Data collection  

Bruker APEXII CCD diffractometer
Absorption correction: multi-scan (*SADABS*; Sheldrick, 2008*a* ▶) *T*_min_ = 0.661, *T*_max_ = 0.747
15568 measured reflections
4902 independent reflections
4331 reflections with *I* > 2σ(*I*)
*R*_int_ = 0.030

#### Refinement  

*R*[*F* ^2^ > 2σ(*F* ^2^)] = 0.041
*wR*(*F* ^2^) = 0.111
*S* = 1.04
4902 reflections
158 parameters
1 restraint
H-atom parameters constrained
Δρ_max_ = 0.30 e Å^−3^
Δρ_min_ = −0.18 e Å^−3^

### 

Data collection: *APEX2* (Bruker, 2012 ▶); cell refinement: *SAINT* (Bruker, 2012 ▶); data reduction: *SAINT*; program(s) used to solve structure: *SIR97* (Altomare *et al.*, 1999 ▶); program(s) used to refine structure: *SHELXL2013* (Sheldrick, 2008*b* ▶); molecular graphics: *ORTEPIII* (Burnett & Johnson, 1996 ▶), *ORTEP-3 for Windows* (Farrugia, 2012 ▶) and *Mercury* (Macrae *et al.*, 2006 ▶); software used to prepare material for publication: *SHELXL2013*.

## Supplementary Material

Crystal structure: contains datablock(s) I, New_Global_Publ_Block. DOI: 10.1107/S1600536813034041/fy2107sup1.cif

Structure factors: contains datablock(s) I. DOI: 10.1107/S1600536813034041/fy2107Isup2.hkl

Supporting information file. DOI: 10.1107/S1600536813034041/fy2107Isup3.cml

Additional supporting information:  crystallographic information; 3D view; checkCIF report

## Figures and Tables

**Table 1:** Hydrogen-bond geometry (Å, °)

*D*—H⋯*A*	*D*—H	H⋯*A*	*D*⋯*A*	*D*—H⋯*A*
C2—H2*B*⋯O1^i^	0.99	2.60	3.541 (2)	160

Symmetry code: (i) 
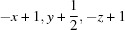
.

**Fig. 1.**
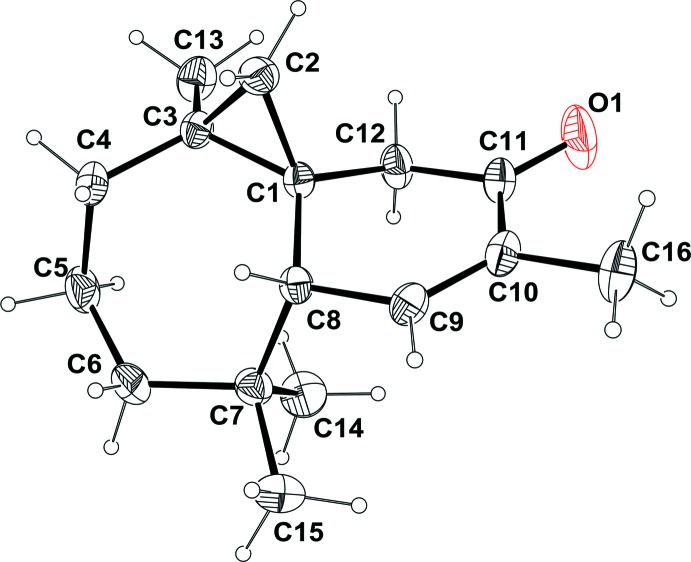
The asymmetric unit of (I) with the atom-labelling scheme. Displacement ellipsoids are drawn at the 50% probability level. H atoms are represented as small spheres of arbitrary radii.

**Fig. 2.**
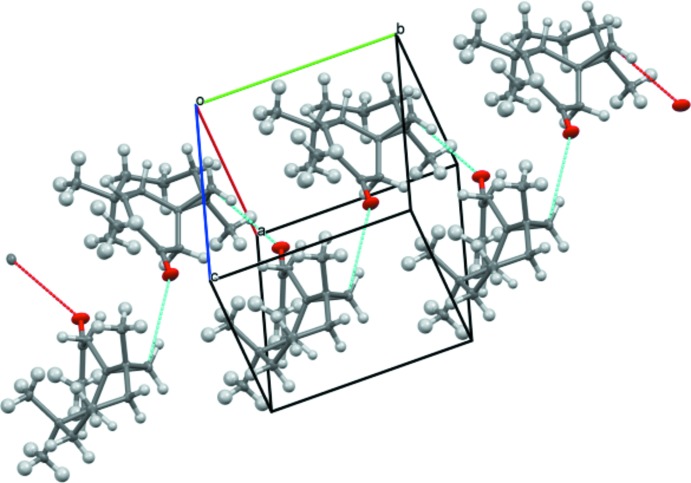
Partial packing view (Mercury) showing the weak C—H···O interaction as dashed lines.

## supplementary crystallographic information

### 1. Comment

α,β-unsaturated ketones are versatile intermediates in organic synthesis, since they are important precursors in the synthesis of enaminones (Auhmani *et al.*, 2002), prodrugs (Danyang *et al.*, 2007) and natural compounds (Tetsuhiro *et al.*, 2003).

With the aim of preparing a new α,β-unsaturated ketone with sesquiterpenic skeleton, we report here the synthesis of (1*R*,3S,8*R*)-3,7,7,10-tetramethyltricyclo[6.4.0.0^1.3^]dodec-9-en-11-one, 1. (1*S*,3S,8*R*)-3,7,7,10-tetramethyltricyclo[6.4.0.0^1,3^]dodec-9-ene, 2, was treated with *N*-bromosuccinimide (NBS). The reaction was conducted at 0°C to provide the α,β-unsaturated ketone 1 as colorless crystals in 62% yield. Its structure was characterized by its mass and NMR spectroscopic data. Furthermore, an X-ray single-crystal structure analysis allowed its complete identification as (1*R*,3S,8*R*)-3,7,7,10-tetramethyltricyclo[6.4.0.0^1.3^]dodec-9-en-11-one.

A view of the molecule is represented in Fig. 1. As observed in related compounds (Gassman & Gorman, 1990; Lassaba *et al.*, 1997; Benharref *et al.*, 2012), each molecule is built up from two fused six-and seven-membered rings. The six-membered ring has roughly half-chair conformation with the puckering parameters: Q = 0.4166 (11) Å, spherical polar angle θ = 124.02 (15)° and φ = 170.57 (19)° (Spek, 2009; Cremer & Pople, 1975), whereas the seven-membered ring displays a chair conformation with a total puckering amplitude of 0.7919 (11) Å (Evans & Boeyens, 1989).

There is a weak C—H···O hydrogen bond, which builds up a zigzag-like chain developing along the *b* axis (Table 1, Fig. 2)

### 2. Experimental

To a cooled (0°C) solution of (1*S*,3S,8*R*)-3,7,7,10-tetramethyl tricyclo[6.4.0.0^1,3^]dodec-9-ene 2 (4.6 mmol) in 50 ml of a solvent mixture THF/H_2_O (4/1, *v*/*v*), NBS (9.16 mmol) was added in small portions. The mixture was kept under stirring at 0°C for two hours. After completion of the reaction, 15% sodium hydrogenocarbonate solution was added and the reaction mixture was taken up in ether, dried over anhydrous sodium sulfate, and evaporated. The crude product was purified by silica gel chromatography (230–400 mesh) using hexane/ethyl acetate (95:5) as eluent to give (1*R*,3S,8*R*)-3,7,7,10-tetramethyltricyclo[6.4.0.0^1.3^]dodec-9-en-11-one, 1 (2.85 mmol) in 62% yield. X-ray quality crystals were obtained by slow solvent evaporation from an isohexane solution of the title compound.

### 3. Refinement

All H atoms attached to C atoms were fixed geometrically and treated as riding with C—H = 0.99 Å (methylene), 0.98 Å (methyl), 0.95 Å (methine) with *U*_iso_(H) = 1.2*U*_eq_(CH and CH_2_) or *U*_iso_(H) = 1.5*U*_eq_(CH_3_).

In the absence of significant anomalous scattering, the absolute configuration could not be reliably determined and any references to the Flack parameter were removed.

The 0 0 1 reflection appears affected by the beam stop and it has been removed.

### Crystal data

**Table:**

C_16_H_24_O	*F*(000) = 256
*M**_r_* = 232.35	*D*_x_ = 1.134 Mg m^−^^3^
Monoclinic, *P*2_1_	Mo *K*α radiation, λ = 0.71073 Å
Hall symbol: P 2yb	Cell parameters from 7217 reflections
*a* = 6.4379 (2) Å	θ = 3.0–33.0°
*b* = 7.8889 (3) Å	µ = 0.07 mm^−^^1^
*c* = 13.5122 (5) Å	*T* = 180 K
β = 97.430 (2)°	Plate, colourless
*V* = 680.49 (4) Å^3^	0.38 × 0.23 × 0.08 mm
*Z* = 2	

### Data collection

**Table:**

Bruker APEXII CCD diffractometer	4902 independent reflections
Radiation source: sealed tube	4331 reflections with *I* > 2σ(*I*)
Graphite monochromator	*R*_int_ = 0.030
φ and ω scans	θ_max_ = 33.2°, θ_min_ = 3.0°
Absorption correction: multi-scan (*SADABS*; Sheldrick, 2008*a*)	*h* = −9→9
*T*_min_ = 0.661, *T*_max_ = 0.747	*k* = −12→12
15568 measured reflections	*l* = −20→20

### Refinement

**Table:**

Refinement on *F*^2^	1 restraint
Least-squares matrix: full	Hydrogen site location: inferred from neighbouring sites
*R*[*F*^2^ > 2σ(*F*^2^)] = 0.041	H-atom parameters constrained
*wR*(*F*^2^) = 0.111	*w* = 1/[σ^2^(*F*_o_^2^) + (0.0667*P*)^2^ + 0.0218*P*] where *P* = (*F*_o_^2^ + 2*F*_c_^2^)/3
*S* = 1.04	(Δ/σ)_max_ = 0.001
4902 reflections	Δρ_max_ = 0.30 e Å^−^^3^
158 parameters	Δρ_min_ = −0.18 e Å^−^^3^

### Special details

**Table:**

Geometry. All e.s.d.'s (except the e.s.d. in the dihedral angle between two l.s. planes) are estimated using the full covariance matrix. The cell e.s.d.'s are taken into account individually in the estimation of e.s.d.'s in distances, angles and torsion angles; correlations between e.s.d.'s in cell parameters are only used when they are defined by crystal symmetry. An approximate (isotropic) treatment of cell e.s.d.'s is used for estimating e.s.d.'s involving l.s. planes.

### Fractional atomic coordinates and isotropic or equivalent isotropic displacement parameters (Å^2^)

**Table:**

	*x*	*y*	*z*	*U*_iso_*/*U*_eq_	
C1	0.31651 (18)	0.79421 (18)	0.28074 (9)	0.0192 (2)	
C2	0.2552 (2)	0.98024 (19)	0.27484 (11)	0.0255 (3)	
H2A	0.1104	1.0088	0.2457	0.031*	
H2B	0.3159	1.0555	0.3297	0.031*	
C3	0.40116 (18)	0.91018 (18)	0.20572 (9)	0.0213 (3)	
C4	0.3155 (2)	0.8894 (2)	0.09629 (10)	0.0261 (3)	
H4A	0.3706	0.9821	0.0577	0.031*	
H4B	0.1611	0.9005	0.0888	0.031*	
C5	0.3716 (2)	0.7200 (2)	0.05222 (11)	0.0318 (3)	
H5A	0.5148	0.6876	0.0819	0.038*	
H5B	0.3727	0.7338	−0.0205	0.038*	
C6	0.2204 (3)	0.5769 (2)	0.07006 (11)	0.0322 (3)	
H6A	0.2565	0.4778	0.0308	0.039*	
H6B	0.0780	0.6135	0.0417	0.039*	
C7	0.2086 (2)	0.51533 (19)	0.17792 (10)	0.0244 (3)	
C8	0.14660 (17)	0.66525 (17)	0.24508 (9)	0.0191 (2)	
H8	0.0359	0.7304	0.2023	0.023*	
C9	0.04465 (19)	0.60496 (19)	0.33343 (10)	0.0236 (3)	
H9	−0.0870	0.5499	0.3191	0.028*	
C10	0.1211 (2)	0.6213 (2)	0.42964 (11)	0.0263 (3)	
C11	0.3324 (2)	0.6937 (2)	0.45837 (10)	0.0271 (3)	
C12	0.4560 (2)	0.7448 (2)	0.37599 (10)	0.0263 (3)	
H12A	0.5471	0.6493	0.3614	0.032*	
H12B	0.5474	0.8418	0.3988	0.032*	
C13	0.6305 (2)	0.9621 (2)	0.22205 (12)	0.0310 (3)	
H13A	0.6697	0.9941	0.2921	0.047*	
H13B	0.6521	1.0589	0.1791	0.047*	
H13C	0.7175	0.8669	0.2056	0.047*	
C14	0.4145 (2)	0.4307 (2)	0.22037 (13)	0.0352 (4)	
H14A	0.4383	0.3308	0.1802	0.053*	
H14B	0.4072	0.3960	0.2895	0.053*	
H14C	0.5301	0.5110	0.2186	0.053*	
C15	0.0350 (3)	0.3801 (2)	0.16921 (14)	0.0372 (4)	
H15A	0.0327	0.3251	0.2341	0.056*	
H15B	0.0626	0.2950	0.1197	0.056*	
H15C	−0.1007	0.4339	0.1483	0.056*	
C16	0.0016 (3)	0.5678 (3)	0.51319 (14)	0.0403 (4)	
H16A	−0.1322	0.5173	0.4852	0.060*	
H16B	−0.0246	0.6670	0.5534	0.060*	
H16C	0.0839	0.4843	0.5553	0.060*	
O1	0.4059 (2)	0.7093 (2)	0.54568 (8)	0.0492 (4)	

### Atomic displacement parameters (Å^2^)

**Table:**

	*U*^11^	*U*^22^	*U*^33^	*U*^12^	*U*^13^	*U*^23^
C1	0.0190 (4)	0.0222 (6)	0.0168 (5)	−0.0001 (4)	0.0033 (4)	0.0009 (4)
C2	0.0287 (6)	0.0230 (7)	0.0263 (6)	0.0003 (5)	0.0095 (5)	−0.0021 (5)
C3	0.0214 (5)	0.0230 (7)	0.0202 (6)	0.0001 (4)	0.0055 (4)	0.0023 (5)
C4	0.0300 (6)	0.0289 (7)	0.0199 (6)	0.0033 (5)	0.0047 (4)	0.0061 (5)
C5	0.0407 (7)	0.0376 (9)	0.0189 (6)	0.0035 (6)	0.0100 (5)	−0.0003 (6)
C6	0.0446 (7)	0.0312 (8)	0.0208 (7)	0.0009 (6)	0.0043 (5)	−0.0060 (6)
C7	0.0277 (5)	0.0225 (7)	0.0227 (6)	0.0030 (5)	0.0019 (4)	−0.0019 (5)
C8	0.0175 (4)	0.0214 (6)	0.0181 (5)	0.0022 (4)	0.0015 (4)	0.0013 (5)
C9	0.0205 (5)	0.0246 (7)	0.0263 (6)	0.0004 (5)	0.0057 (4)	0.0040 (5)
C10	0.0284 (6)	0.0277 (7)	0.0242 (6)	0.0020 (5)	0.0082 (5)	0.0070 (5)
C11	0.0308 (6)	0.0311 (8)	0.0190 (6)	0.0015 (5)	0.0019 (4)	0.0064 (5)
C12	0.0225 (5)	0.0371 (8)	0.0185 (6)	−0.0035 (5)	0.0000 (4)	0.0033 (5)
C13	0.0240 (5)	0.0370 (9)	0.0326 (7)	−0.0047 (5)	0.0061 (5)	0.0056 (6)
C14	0.0369 (7)	0.0299 (8)	0.0390 (8)	0.0146 (6)	0.0056 (6)	−0.0002 (6)
C15	0.0438 (8)	0.0276 (8)	0.0393 (8)	−0.0068 (6)	0.0020 (6)	−0.0064 (7)
C16	0.0423 (7)	0.0500 (11)	0.0312 (8)	−0.0022 (7)	0.0150 (6)	0.0136 (7)
O1	0.0509 (6)	0.0765 (11)	0.0185 (5)	−0.0110 (7)	−0.0021 (4)	0.0070 (6)

### Geometric parameters (Å, º)

**Table:**

C1—C3	1.5181 (18)	C8—H8	1.0000
C1—C2	1.519 (2)	C9—C10	1.336 (2)
C1—C12	1.5220 (18)	C9—H9	0.9500
C1—C8	1.5255 (18)	C10—C11	1.4796 (19)
C2—C3	1.5123 (17)	C10—C16	1.5065 (19)
C2—H2A	0.9900	C11—O1	1.2192 (17)
C2—H2B	0.9900	C11—C12	1.5048 (18)
C3—C4	1.5186 (19)	C12—H12A	0.9900
C3—C13	1.5207 (17)	C12—H12B	0.9900
C4—C5	1.525 (2)	C13—H13A	0.9800
C4—H4A	0.9900	C13—H13B	0.9800
C4—H4B	0.9900	C13—H13C	0.9800
C5—C6	1.530 (2)	C14—H14A	0.9800
C5—H5A	0.9900	C14—H14B	0.9800
C5—H5B	0.9900	C14—H14C	0.9800
C6—C7	1.548 (2)	C15—H15A	0.9800
C6—H6A	0.9900	C15—H15B	0.9800
C6—H6B	0.9900	C15—H15C	0.9800
C7—C14	1.528 (2)	C16—H16A	0.9800
C7—C15	1.538 (2)	C16—H16B	0.9800
C7—C8	1.5733 (18)	C16—H16C	0.9800
C8—C9	1.5115 (17)		
			
C3—C1—C2	59.73 (9)	C1—C8—C7	117.29 (9)
C3—C1—C12	119.71 (10)	C9—C8—H8	105.6
C2—C1—C12	114.38 (12)	C1—C8—H8	105.6
C3—C1—C8	119.72 (11)	C7—C8—H8	105.6
C2—C1—C8	117.19 (10)	C10—C9—C8	126.53 (12)
C12—C1—C8	114.63 (11)	C10—C9—H9	116.7
C3—C2—C1	60.10 (9)	C8—C9—H9	116.7
C3—C2—H2A	117.8	C9—C10—C11	120.23 (11)
C1—C2—H2A	117.8	C9—C10—C16	122.86 (13)
C3—C2—H2B	117.8	C11—C10—C16	116.91 (14)
C1—C2—H2B	117.8	O1—C11—C10	121.43 (13)
H2A—C2—H2B	114.9	O1—C11—C12	120.82 (13)
C2—C3—C1	60.17 (9)	C10—C11—C12	117.73 (12)
C2—C3—C4	117.69 (11)	C11—C12—C1	112.55 (10)
C1—C3—C4	117.95 (11)	C11—C12—H12A	109.1
C2—C3—C13	118.74 (12)	C1—C12—H12A	109.1
C1—C3—C13	119.42 (11)	C11—C12—H12B	109.1
C4—C3—C13	113.19 (11)	C1—C12—H12B	109.1
C3—C4—C5	113.60 (12)	H12A—C12—H12B	107.8
C3—C4—H4A	108.8	C3—C13—H13A	109.5
C5—C4—H4A	108.8	C3—C13—H13B	109.5
C3—C4—H4B	108.8	H13A—C13—H13B	109.5
C5—C4—H4B	108.8	C3—C13—H13C	109.5
H4A—C4—H4B	107.7	H13A—C13—H13C	109.5
C4—C5—C6	113.40 (11)	H13B—C13—H13C	109.5
C4—C5—H5A	108.9	C7—C14—H14A	109.5
C6—C5—H5A	108.9	C7—C14—H14B	109.5
C4—C5—H5B	108.9	H14A—C14—H14B	109.5
C6—C5—H5B	108.9	C7—C14—H14C	109.5
H5A—C5—H5B	107.7	H14A—C14—H14C	109.5
C5—C6—C7	119.33 (13)	H14B—C14—H14C	109.5
C5—C6—H6A	107.5	C7—C15—H15A	109.5
C7—C6—H6A	107.5	C7—C15—H15B	109.5
C5—C6—H6B	107.5	H15A—C15—H15B	109.5
C7—C6—H6B	107.5	C7—C15—H15C	109.5
H6A—C6—H6B	107.0	H15A—C15—H15C	109.5
C14—C7—C15	108.17 (13)	H15B—C15—H15C	109.5
C14—C7—C6	110.16 (12)	C10—C16—H16A	109.5
C15—C7—C6	105.63 (12)	C10—C16—H16B	109.5
C14—C7—C8	112.60 (11)	H16A—C16—H16B	109.5
C15—C7—C8	109.25 (11)	C10—C16—H16C	109.5
C6—C7—C8	110.74 (12)	H16A—C16—H16C	109.5
C9—C8—C1	109.11 (10)	H16B—C16—H16C	109.5
C9—C8—C7	112.79 (12)		
			
C12—C1—C2—C3	111.44 (11)	C3—C1—C8—C7	67.20 (16)
C8—C1—C2—C3	−110.21 (12)	C2—C1—C8—C7	136.14 (12)
C1—C2—C3—C4	108.01 (14)	C12—C1—C8—C7	−85.61 (13)
C1—C2—C3—C13	−109.34 (14)	C14—C7—C8—C9	−80.64 (14)
C12—C1—C3—C2	−102.54 (14)	C15—C7—C8—C9	39.58 (15)
C8—C1—C3—C2	106.03 (12)	C6—C7—C8—C9	155.52 (11)
C2—C1—C3—C4	−107.58 (13)	C14—C7—C8—C1	47.41 (16)
C12—C1—C3—C4	149.87 (13)	C15—C7—C8—C1	167.63 (12)
C8—C1—C3—C4	−1.55 (17)	C6—C7—C8—C1	−76.43 (14)
C2—C1—C3—C13	108.24 (15)	C1—C8—C9—C10	−17.09 (19)
C12—C1—C3—C13	5.7 (2)	C7—C8—C9—C10	115.13 (15)
C8—C1—C3—C13	−145.73 (13)	C8—C9—C10—C11	−4.7 (2)
C2—C3—C4—C5	−134.89 (13)	C8—C9—C10—C16	175.72 (16)
C1—C3—C4—C5	−65.84 (15)	C9—C10—C11—O1	−179.74 (16)
C13—C3—C4—C5	80.48 (16)	C16—C10—C11—O1	−0.1 (2)
C3—C4—C5—C6	84.92 (16)	C9—C10—C11—C12	−0.8 (2)
C4—C5—C6—C7	−65.63 (18)	C16—C10—C11—C12	178.82 (15)
C5—C6—C7—C14	−66.50 (18)	O1—C11—C12—C1	−153.30 (16)
C5—C6—C7—C15	176.90 (14)	C10—C11—C12—C1	27.8 (2)
C5—C6—C7—C8	58.73 (16)	C3—C1—C12—C11	156.43 (13)
C3—C1—C8—C9	−163.00 (11)	C2—C1—C12—C11	88.68 (15)
C2—C1—C8—C9	−94.06 (13)	C8—C1—C12—C11	−50.76 (17)
C12—C1—C8—C9	44.19 (14)		

### Hydrogen-bond geometry (Å, º)

**Table:**

*D*—H···*A*	*D*—H	H···*A*	*D*···*A*	*D*—H···*A*
C2—H2*B*···O1^i^	0.99	2.60	3.541 (2)	160

Symmetry code: (i) −*x*+1, *y*+1/2, −*z*+1.
